# Sustained Regression of Hydroxycarbamide Induced Actinic Keratoses after Switching to Anagrelide

**DOI:** 10.1155/2018/2874012

**Published:** 2018-03-27

**Authors:** Georgios Gaitanis, Dora Gougopoulou, Eleni Kapsali, Ioannis D. Bassukas

**Affiliations:** ^1^Department of Skin and Venereal Diseases, Faculty of Medicine, School of Health Sciences, University of Ioannina, Ioannina, Greece; ^2^Hematology Clinic, Department of Internal Medicine, Faculty of Medicine, School of Health Sciences, University of Ioannina, Ioannina, Greece

## Abstract

Hydroxycarbamide (HC) is the first-line treatment for certain myeloproliferative neoplasms, such as polycythemia vera and essential thrombocytosis (ET). In a subset of these patients long-term treatment with HC can result in the development of confluent actinic keratoses (AK) followed by invasive keratinocytic carcinomas (“squamous dysplasia”), preferentially on sun-exposed skin. Discontinuation or dose reduction of HC may result in partial improvement. A 59-year-old farmer after 14 years on HC (2 gr/d) and acetylsalicylic acid (100 mg/d) for ET, was referred for numerous, hyperkeratotic AK on face, scalp, and hands that could not be controlled with repeated (*N* = 15) cryosurgery sessions in the previous 3 years. Acitretin (0.32 mg/kg daily) and topical treatments (cryosurgery with ingenol mebutate) were initiated with only marginal improvement after 3 months. Acitretin dose was doubled and HC was switched to anagrelide (0.5 mg twice daily). Within a month the AK load regressed significantly and, at 3 months follow-up, complete clinical remission was achieved and acitretin was discontinued. Twenty months later the patient is clear from AK. In conclusion, the impressive and sustainable AK remission under anagrelide draws attention to a possible role of the phosphodiesterase 3 pathway, the major pharmacological target of anagrelide, as a potential therapeutic target for keratinocytic cancers.

## 1. Introduction

Hydroxycarbamide (HC) is recommended as the first-line treatment modality for the management of patients within the spectrum of certain myeloproliferative neoplasms, as polycythemia vera and essential thrombocytosis (ET) [1, 2]. HC is a potent inhibitor of the ribonucleotide reductase and slows down the rate of DNA replication decelerating cell cycle progression at the G1/S phase transition point and elongating the S phase both* in vitro* and* in vivo* [3]. However, this exposes cell populations, like epidermal keratinocytes, to the action of carcinogens, as ensuing mutations are established prior to DNA replication and cell division [4]. A subset of patients receiving HC for the above hematologic conditions will develop multiple actinic keratoses (AK) and keratinocytic carcinomas in sun-exposed skin regions (“squamous dysplasia”) [5]. This is a serious and therapeutically challenging side-effect [6], which may partly improve after HC discontinuation or dose reduction [7]. Anagrelide, on the other hand, is a phosphodiesterase 3 (PDE 3) inhibitor that is recommended as a 3rd-line treatment for ET after interferon-*α* (IFN-*α*) and busulfan [2].

## 2. Case Presentation

**Figure 1:**
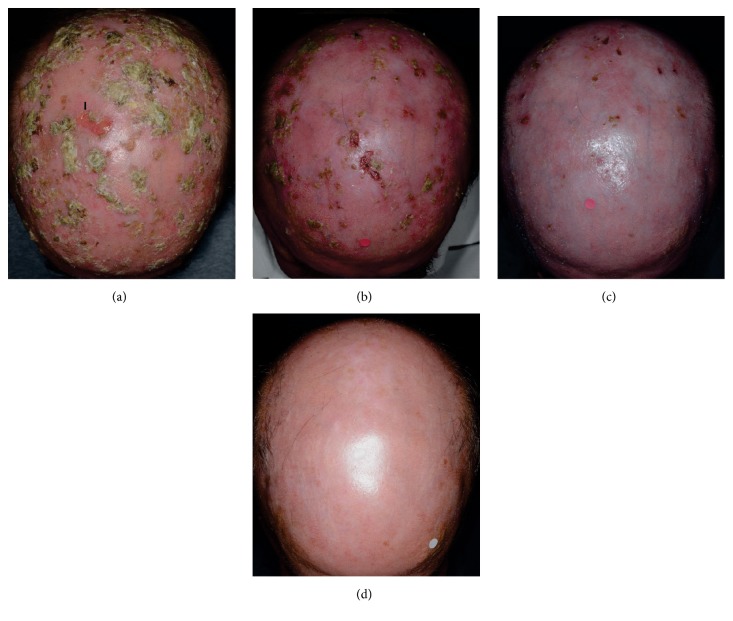
Complete and sustained remission of extensive hydroxycarbamide-associated skin carcinogenesis fields after switching to anagrelide: exemplary presentation of the alterations on the balding scalp skin. (a) At presentation numerous, confluent, hypertrophic actinic keratoses cover almost the entire scalp. Removal of the hyperkeratosis revealed an oozing erosion (arrow). (b) A significant load of actinic keratoses persists 3 months after onset of treatment with cryosurgery plus ingenol mebutate (7 treatment cycles) and daily 0.32 mg/kg b.w. acitretin. At that point acitretin dose was doubled (0.64 mg/kg b.w./d) and the patient was switched from hydroxycarbamide to anagrelide. (c) Impressive improvement after one month in the last treatment scheme (anagrelide and acitretin, no topical treatments). (d) Sustainable “clearance” of the field 20 months after discontinuation of acitretin and still on anagrelide: complete regression of existing and no development of new actinic keratoses.

A 59-year-old farmer on HC for ET was referred due to multiple, hyperkeratotic AK accumulating in chronically sun-exposed skin areas: the hands, ears, almost the entire balding scalp, and large portions of the face, accompanied by severe actinic cheilitis. The majority of the lesions, especially on the scalp, were covered by a hyperkeratotic crust that could be removed relatively easy, revealing an oozing erosion (Figure 1(a)). ET was diagnosed 14 years earlier and was treated since with HC (2 gr/d) and acetylsalicylic acid (100 mg/d). The medical history of the patient was also remarkable for hyperlipidemia on simvastatin (20 mg/d) and fenofibrate (145 mg/d), arterial hypertension on atenolol (50 mg/d), and gastroesophageal reflux on rabeprazole (20 mg/d). AK burden had progressively expanded in the past 3 years. During this period, repeated sessions of cryosurgery (*N* = 15) failed to control existing lesions while new ones continued to appear. Biopsies from a hyperkeratotic scalp lesion and from the lower lip confirmed AK and actinic cheilitis, respectively. Due to the hyperlipidemia, the patient was initiated on a moderate acitretin dose (0.32 mg/kg body weight daily). Concurrently, skin segments (25 cm^2^ each) with HC induced “squamous dysplasia” [5] were treated topically with the combination of cryosurgery sessions (liquid N_2_, open spray, 2 cycles of 10 sec each, applied in quarters of the segment) and, starting on the same day, application of ingenol mebutate gel as per manufacturer's recommendations. After 3 months on this treatment (7 treatment cycles) the AK load had only marginally improved (Figure 1(b)). At this time the dose of acitretin was doubled and HC was switched to anagrelide (0.5 mg twice daily). Already within a month after the latter treatment adaptation an impressive regression of the AK load was evident (Figure 1(c)). At the 3-month follow-up, complete AK remission was recorded and acitretin was discontinued. The temporal regression of lesions was noted concurrently in all affected skin regions and most probably represents sustained remission of the sum of carcinogenesis fields of this occupationally heavily sun-exposed patient. To date, 20 months later, no new AKs have developed (Figure 1(d)) and as we could not identify any clinical signs of AK to sample, a confirmatory biopsy was not performed (Figure 1(d)).

Medline search (January 15, 2018) with conjunction of the terms “‘hydroxycarbamide' AND ‘skin', ‘hydroxycarbamide' AND (‘actinic' OR ‘keratosis')” returned 436 and 34 articles, respectively. Extensive search within these articles for comparable cases to the present one located only two relevant reports: the first one [8] describes a 59-year-old man with ET under HC who was switched to anagrelide due to the development of multiple squamous cell carcinomas. The first six months after treatment revision a multitude of skin cancers were continued to be encountered (3 basal cell carcinomas, 4 squamous cell carcinomas, and 10 AK), yet no new lesions developed subsequently (total follow-up period is not reported). The second report [9], probably more relevant to the present case, described a 50-year-old female with ET under HC for 26 years. After she had developed extensive AK fields resistant to multiple local treatment cycles for 6 years, she was switched to anagrelide in February 2017 with subsequent rapid improvement.

## 3. Discussion

Thus, including the present case, 3 patients have notable response of squamous dysplasia after switching of HC to anagrelide. In our case, the remission lasts already 20 months after drug switching (the longest follow-up period among reported patients). This sustainable effect indicates the existence of a protective action of anagrelide against the development of AK. The impressive clinical improvement of this patient contrasts the relative scarcity of reported cases with a similar outcome and raises the question of whether this was merely an isolated phenomenon or could represent a still underreported effect. To our opinion the latter explanation is probably more relevant for various reasons. Not all patients under HC will progress to the development of squamous dysplasia. The increase in the rates of nonmelanoma skin cancer development after significant cumulative HC doses is approximately 22% [5]. This points towards the existence of a susceptible subpopulation within HC treated patients that will develop overt “squamous dysplasia.” As already mentioned, when these patients will require substitution of HC, the recommended 2nd-line medications are either IFN-*α* or busulfan, followed by anagrelide as a 3rd-line treatment option [2]. Both IFN-*α* and busulfan have distinct effects on skin cancers and skin carcinogenesis fields: IFN-*α* is employed as a treatment modality in selected cases of keratinocytic cancers [10], while busulfan increases the risk for the development of these neoplasms [6]. Thus, anagrelide will be used in a relative small proportion of patients with “squamous dysplasia” directly after HC to permit recording and attributing possible antineoplastic effects to this medication. It is worth noting that the only intricacy restricting exclusive attribution to anagrelide of the impressive AK clearance effect in our patient is that he received it together with acitretin for 3 months. A still unrecognized synergistic action of both substances against “squamous dysplasia” cannot be excluded, as well as the exact role of HC discontinuation in the regression of the induced AK fields. Retinoids do have a recognized action against carcinogenesis fields and are recommended, for example, in the treatment of multiple AK developing in transplanted patients under immun*ο*suppression [11]. Yet, their prophylactic effect stops after discontinuation. On the other hand, PDE3A, the target of anagrelide, is highly expressed in epithelial cancer cell lines, including lung, colon, and cervical cancer [12] and its targeted inhibition is evaluated for redirection of PDE3 inhibitors in anticancer treatment [13] as well as an intense search for new ones [14]. PDE3A is expressed ~40% more in sun-exposed (tibial) compared to sun-protected skin [14] underscoring the need to evaluate its expression in keratinocytic carcinomas, including AK. Likewise, in an experimental mouse model, cilostamide, a PDE3 inhibitor, increased apoptosis in UV damaged keratinocytes by 29%, while other PDE inhibitors (PDE2 inhibitors) demonstrated even more pronounced effects [15].

In conclusion, the rapid and sustained regression of HC induced AK fields draws attention to a possible role of the PDE 3 pathway, the major pharmacological target of anagrelide, as a promising intervention for keratinocytic cancers that warrants further investigation.
